# Segment-specific association between cervical pillar hyperplasia (CPH) and degenerative joint disease (DJD)

**DOI:** 10.1186/1746-1340-14-21

**Published:** 2006-09-13

**Authors:** Maja Stupar, Cynthia K Peterson

**Affiliations:** 1 Division of Clinical Education, Canadian Memorial Chiropractic College, 6100 Leslie Street, Toronto, ON M2H 3J1, Canada; 2 Department of Radiology, Canadian Memorial Chiropractic College, 6100 Leslie Street, Toronto, ON M2H 3J1, Canada

## Abstract

**Background:**

Cervical pillar hyperplasia (CPH) is a recently described phenomenon of unknown etiology and clinical significance. Global assessment of pillar hyperplasia of the cervical spine as a unit has not shown a relationship with degenerative joint disease, but a more sensible explanation of the architectural influence of CPH on cervical spine biomechanics may be segment-specific.

**Objective:**

The objective of this study was to determine the level of association between degenerative joint disease (DJD) and cervical pillar hyperplasia (CPH) in an age- and gender-matched sample on a [cervical spine] by-level basis.

**Research Methods:**

Two-hundred and forty radiographs were collected from subjects ranging in age between 40 and 69 years. The two primary outcome measures used in the study were the segmental presence/absence of cervical pillar hyperplasia from C3 to C6, and segment-specific presence/absence of degenerative joint disease from C1 to C7. Contingency Coefficients, at the 5% level of significance, at each level, were used to determine the strength of the association between CPH and DJD. Odds Ratios (OR) with their 95% Confidence Intervals (95% CI) were also calculated at each level to assess the strength of the association.

**Results:**

Our study suggests that an approximately two-to-one odds, or a weak-to-moderate correlation, exists at C4 and C5 CPH and adjacent level degenerative disc disease (DDD); with the strongest (overall) associations demonstrated between C4 CPH and C4–5 DDD and between C5 CPH and C5–6 DDD. Age-stratified results demonstrated a similar pattern of association, even reaching the initially hypothesized OR ≥ 5.0 (95% CI > 1.0) or "moderately-strong correlation of C ≥ .4 (p ≤ .05)" in some age categories, including the 40–44, 50–59, and 60–64 years of age subgroups; these ORs were as follows: OR = 5.5 (95% CI 1.39–21.59); OR = 6.7 (95% CI 1.65–27.34); and OR = 5.3 (95% CI 1.35–21.14), respectively.

**Conclusion:**

Our results suggest that CPH has around two-to-one odds, that is, only a weak-to-moderate association with the presence of DJD (DDD component) at specific cervical spine levels; therefore, CPH may be but one of several factors that contributes (to a clinically important degree) to the development of DJD at specific levels in the cervical spine.

## Background

Cervical pillar hyperplasia (CPH) is a radiological finding which first made its appearance in the literature less than 30 years ago [1-4]. Its etiology and clinical significance are presently unknown; nevertheless, studies have shown that CPH is a frequently overlooked etiology for the loss of the cervical lordosis [2,3]. While these findings were disputed by several authors [5-9], other consequences of cervical pillar hyperplasia are not known at the present time. It has been theorized that the architectural difference that the presence of hyperplasia introduces into the cervical pillar may cause segmental biomechanical changes and may lead to a higher prevalence of degenerative joint disease (DJD) at the hyperplastic or adjacent cervical levels. The clinical significance of this phenomenon, if found to be related to degenerative joint disease, should prompt an astute clinician into evaluating the articular pillars on all cervical spine radiographs – particularly because there could be a chance that the patient may develop degeneration at the specific cervical levels and may experience associated neck pain. The architecture of the cervical pillars cannot be modified by conservative therapy; therefore, clinicians should be aware that some of the symptoms may be attributed to degeneration and may influence the expected prognosis of the management of neck pain in those particular patients.

Currently, it is unknown whether the architecture of the articular pillars has a clinically important effect on segmental biomechanics and subsequent degeneration. The axis around which segmental flexion/extension occurs is principally influenced by the orientation of the facet joint in relation to the horizontal plane [10,11]. A more horizontal facet in comparison to the plane of the superior endplate will shift the instantaneous center of rotation anteriorly, resulting in an increased load on the intervertebral disc (with changes in lateral flexion motion) and an increased risk of anterolisthesis [10,12-14]. A straightening of the cervical curve, possibly caused by CPH [2,3], results in a redistribution of the loads, favouring the facet joints, and therefore increasing the load on the associated intervertebral disc. Although this has only been demonstrated in a mechanical model, the evidence to-date suggests that an increase of the stress-load on the intervertebral disc locally may enhance the degenerative process in the intervertebral compartment [15]. The possibility that a change in architecture may change the biomechanics of the cervical spine [16] has led to the hypothesis that individuals who have CPH may be predisposed to more biomechanical stress, as is involved in the predominantly accepted theory of the development of degenerative joint disease.

In our preliminary study, although we found no clinically important difference between the global presence/absence of cervical pillar hyperplasia and prevalence of DJD, a more sensible explanation of the architectural influence of CPH on cervical spine biomechanics may be segment-specific, meaning that a hyperplastic pillar at a specific cervical level may be related to a higher prevalence of DJD at that specific level and/or one segment above or below. We therefore recommended that follow-up research evaluate the segment-specific contribution of pillar hyperplasia to the development and severity of DJD, because a segmental effect on the biomechanics of the cervical spine is more probable. We assume that the degenerative processes would be a result of the cervical pillar hyperplasia and not the opposite, since CPH has been observed at all ages.

The cervical spine experiences a combination of active mobility and loading stresses [17], and is therefore, a region of the spine that is frequently affected by progressive degenerative processes. These processes lead to the condition called "degenerative disc disease" (DDD) characterized by narrowing of the intervertebral discs, development of osteophytes, intercalary bones and surrounding subchondral sclerosis [18-21]. Similar radiographic findings affecting the facet and uncovertebral joints can also be present in facet arthrosis and uncovertebral arthrosis, respectively [18,19,21].

Several grading systems have been developed to determine the degree of degeneration radiographically, using signs of subchondral sclerosis, joint space irregularity, decreased joint space and anterior and/or posterior osteophyte formation [19,22]. *DJD, osteoarthrosis*, or *cervical spondylosis*, are terms attributed to one or a combination of these findings affecting the disc (DDD), uncovertebral, and facet joints (uncovertebral and facet arthrosis) at a particular spinal segment.

DJD is a common, age-related, multi-factorial condition with several theorized etiologies including metabolic, mechanical, inflammatory, and genetic components [18-20,23]. This condition affects all joints, especially those that experience chronic biomechanical stresses such as frequent repetitive use and strain, previous trauma and frequent weight-bearing [19].

The clinical implications of these degenerative processes may include: limitation of head and neck mobility, with or without pain; possible intervertebral foramen encroachment and central canal stenosis, which can result in nerve root or spinal cord compression (radiculopathy and myelopathy respectively) [20,24,25]; and, although less common, extensive anterior osteophytosis can lead to dysphagia or even vocal fold paralysis [26]. Some controversy exists in the literature with regard to whether radiological findings are related to the patient's symptoms to a clinically important degree. One long-term follow-up study [27] found that patients' symptoms correlate with radiographic findings; however, the majority of authors to-date found only a weak relationship between radiographic degenerative changes and pain [28-31].

The presence of DJD is often confirmed using plain film radiographic findings, with the lateral view being the most informative [20]. The reliability of determining the severity of DJD on plain film radiographs in the cervical spine has not been established, but the reliability of detecting the presence or absence of DJD has a substantial-to-almost-perfect agreement when assessing the presence/absence of intervertebral disc narrowing, osteophyte formation, zygapophyseal joint, and uncinate process degeneration [32].

Since the relationship between cervical spine DJD and CPH has only been studied 'globally' (i.e. CPH was judged to be generally present/absent within the cervical spine as a whole, regardless of whether hyperplastic pillars were detected at one or more levels from C3 to C6), the etiology and clinical relevance of CPH remain unknown. The purpose of this paper, therefore, is to determine if there is a clinically important association (OR ≥ 5.0 and C ≥ .4) between cervical pillar hyperplasia CPH) and degenerative joint disease (DJD) at specific cervical levels, in an age- and gender-matched sample, and how strong this relationship is between the two conditions, on a by level basis.

## Methods

### Research Design

This is an association-etiological type of study design.

### Research Hypothesis

We hypothesized that there would be a clinically important association of C ≥ .4 (p ≤ .05) and/or OR ≥ 5.0 (and where the 95% CI does not include 1.0) between the presence of cervical pillar hyperplasia (CPH) at specific cervical segments and the presence of degenerative joint disease (DJD) at specific cervical levels in an age and gender-matched population of subjects with and without CPH.

### Sample Size Estimate

A sample size estimate method for the C-coefficient specifically, was not directly available, so the Pearson Rho method was used as a proxy. An estimate was performed using the specifications of 80% power, 5% significance, and an estimated correlation coefficient of 0.4, and revealed that a minimum total sample size of 46 radiographs would be required. The 50% prevalence at C6 (approximating the previously reported 46% prevalence of CPH at C5 and C6) was assumed to be adequate to satisfy the required estimated sample size specifications at each level from C3 to C6, even with CPH generally being somewhat less common at C3 than at the C5 and C6 levels [3].

Another *ex post facto *sample size estimate was also performed according to the method of Fleiss [33] for Odds Ratios (OR) in Case Control studies. Using the specifications of 80% power, 95% confidence, and a ratio of controls-to-cases of 0.4, the minimum sample sizes which would render the Odds Ratios (ORs) of 1.9 and 5.0 statistically significant, would be 408 and 80, respectively.

### Procedure

Sets of patient radiographs on file at the HK Lee radiology facility were selected at the Canadian Memorial Chiropractic College in Toronto. Independent Ethics Review Board approval was obtained from the same institution, and permission to access files was obtained from the Dean of Clinical Education, prior to the file selection process. The investigator selecting the cases was blinded to the clinical status of the cases. The inclusion criteria for the radiographs were:

a) the radiographs had to be of good radiological quality (including collimation, penetration, and absence of artifacts);

b) each radiograph had to be of a patient in the age range from 40 to 69 yrs (in order to capture age ranges of subjects from low to high prevalence of DJD);

c) the radiographs could not show evidence of a pathologic condition or abnormality other than signs of osteoarthrosis;

d) the files had to consist of at least three views (considered a set of films): an anteroposterior open mouth (APOM), AP lower cervical and a neutral lateral.

Radiographs were evaluated by the first investigator [MS] until a convenience sample of 240 sets of eligible films was collected: 120 *with *and 120 *without *pillar hyperplasia (CPH) at C6; the 'with' and 'without' CPH films were age- and gender-matched. At the time, it was assumed that this sample would reflect the general adult-population prevalence for cervical pillar hyperplasia of 46%, as reported by Peterson et al [3]. Even if CPH was considered a normal variant due to the high prevalence, it would likely introduce biomechanical stresses at its spinal level. Presence/absence of hyperplasia was evaluated at each level from C3 to C6 in order to categorize the individual cervical segment as 'hyperplastic' or '*not *hyperplastic.' Age was recorded in terms of six 5-yr categories ranging between 40 and 69 years of age. Radiographs were read until each age and gender sub-category contained 10 sets of films. The selected sets of films were coded with chronological numbers, and then manually shuffled to randomize their order. When they were subsequently administered to the two assessors, no identifying information was included in the film package.

**Figure 1:**
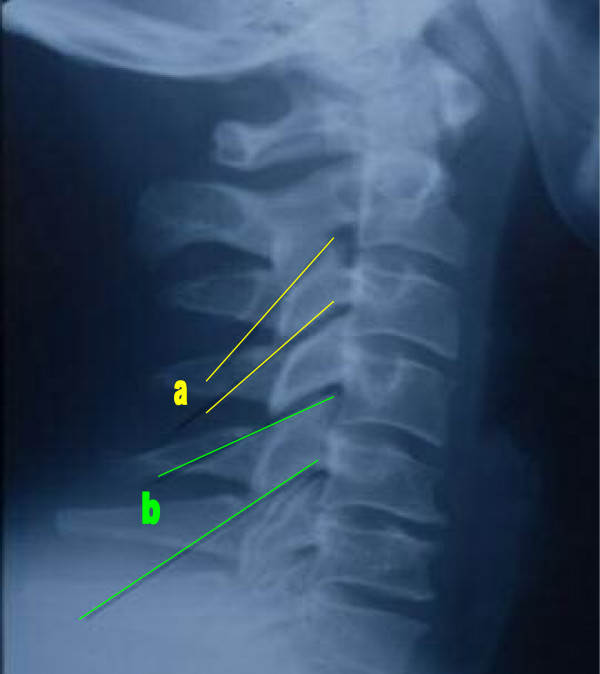
Lateral cervical radiograph demonstrating a normal cervical articular pillar (a) and cervical pillar hyperplasia (b). Previously published by the same authors [4].

The presence of pillar hyperplasia (CPH) was evaluated by the first assessor/investigator [MS] from C3 through C6 by drawing lines along the superior and inferior articular surfaces of each pillar on a neutral lateral cervical radiograph. Lines converging posteriorly designated what was considered 'normal' pillar architecture (Figure 1a). Parallel or posteriorly diverging lines designated what has been defined as 'pillar hyperplasia' (Figure 1b) [2-4]. The reliability of this method of measurement has been determined previously to be moderate-to-substantial [3,4]. The data were categorical (binary nominal) since each pillar was assigned a label of either 'normal' or 'hyperplastic'. The articular pillars of C7 were not evaluated because of their normally 'notched' appearance [34]. Bony hypertrophy from facet arthrosis was not considered a potential confounder because arthrosis normally occurs on the anterior or posterior margins of the facet, not the superior and inferior surfaces used in the definition of CPH.

All 240 sets of films were then reshuffled and re-evaluated for the presence/absence of radiological signs of degenerative joint disease. APOM and AP lower cervical views were used to assess the zygapophyseal joints. Signs of DJD affecting the zygapophyseal joint included subchondral sclerosis, reduction of the joint space, or bony hypertrophy, which result in a disruption of the normally smooth lateral sine curve formed by the external aspect of the articular pillars [18]. Neutral lateral cervical films were used to assess the disc spaces and the presence or absence of anterior or posterior osteophytes. The uncinate processes were not assessed, given that their degenerative changes parallel those of the intervertebral disc [19]. Each radiograph was also evaluated at six levels (from the C1–C2 level to the C6–C7 level) by the second investigator [CP] who was blinded not only to the CPH data collected by the first investigator, but also to any clinical/identifying subject information, and the randomization process. The data were categorical (binary), because each articulation was labelled as either 'normal' or 'showing signs of DJD' (unilateral or bilateral).

### Data Analysis

*Statistica *software was used to determine the association between segment-specific CPH and DJD via the Contingency Coefficient (C-coefficient) at the 5% level of significance, where:

C-coefficient={(Chi-square)/(Chi-square+n})
 MathType@MTEF@5@5@+=feaafiart1ev1aaatCvAUfKttLearuWrP9MDH5MBPbIqV92AaeXatLxBI9gBaebbnrfifHhDYfgasaacH8akY=wiFfYdH8Gipec8Eeeu0xXdbba9frFj0=OqFfea0dXdd9vqai=hGuQ8kuc9pgc9s8qqaq=dirpe0xb9q8qiLsFr0=vr0=vr0dc8meaabaqaciaacaGaaeqabaqabeGadaaakeaacqqGdbWqcqqGTaqlcqqGJbWycqqGVbWBcqqGLbqzcqqGMbGzcqqGMbGzcqqGPbqAcqqGJbWycqqGPbqAcqqGLbqzcqqGUbGBcqqG0baDcqGH9aqpdaGcaaqaaiabcUha7jabcIcaOiabboeadjabbIgaOjabbMgaPjabb2caTiabbohaZjabbghaXjabbwha1jabbggaHjabbkhaYjabbwgaLjabcMcaPiabc+caViabcIcaOiabboeadjabbIgaOjabbMgaPjabb2caTiabbohaZjabbghaXjabbwha1jabbggaHjabbkhaYjabbwgaLjabgUcaRiabb6gaUjabc2ha9jabcMcaPaWcbeaaaaa@61E9@[35].

*SPSS *was used to determine the association between segment-specific CPH and DJD via Odds Ratios (OR) and their 95% Confidence Intervals (95% CI).

## Results

In our sample, the overall CPH prevalence was 11%, 11%, 43% and 50% at C3, C4, C5 and C6, respectively. Our initial assumption of achieving approximately the previously reported 46% prevalence of CPH was true for C5 and C6 but the actual prevalence at C3 and C4 was lower. DDD prevalence, in accordance with previous reports, was highest at the C5–6 and C6–7 levels, with our sample prevalence at C2–3, C3–4, C4–5, C5–6 and C6–7 being at 4%, 15%, 35%, 70%, and 47%, respectively. Prevalence of facet degeneration for the same levels was 22%, 35%, 35%, 35%, and 22%, respectively.

The C-coefficients and ORs for the segmental relationships between cervical pillar hyperplasia (CPH) and degenerative joint disease (DJD) across all age groups (40–69 years) indicate that the strongest associations of statistical significance occurred at C4 and C5; however, these correlation coefficients and ORs were all considerably weaker than the initially proposed clinically important 'moderate' associations represented by C ≥ .4 and OR ≥ 5.0 [Table 1 and Table 2].

**Table 1:** Segmental association between CPH and DJD

**C3 Pillars**						
	C1–2	C2–3 IVD	C2–3 Facet	C3–4 IVD	C3–4 Facet	Sample size

All ages	.05 (p = .467)	.06 (p = .371)	.10 (p = .135)	.01 (p = .927)	.04 (p = .539)	240
40–44	N/A	N/A	.05 (p = .736)	.05 (p = .736)	.12 (p = .426)	40
45–49	.09 (p = .583)	.25 (p = .100)	**.30 (p = .047)**	.02 (p = .875)	.00 (p = 1.00)	40
50–54	.18 (p = .257)	N/A	.08 (p = .588)	.18 (p = .257)	.02 (p = .886)	40
55–59	.12 (p = .426)	.09 (p = .583)	.24 (p = .118)	.17 (p = .271)	.04 (p = .802)	40
60–64	.06 (p = .679)	.04 (p = .816)	.03 (p = .826)	.09 (p = .583)	.17 (p = .261)	40
65–69	.16 (p = .315)	.05 (p = .738)	.01 (p = .962)	.01 (p = .926)	.20 (p = .206)	40

**C4 Pillars**						

	C3–4 IVD	C3–4 Facet	C4–5 IVD	C4–5 Facet	Sample size	

All ages	.07 (p = .252)	.06 (p = .338)	**.16 (p = .012)**	.09 (p = .164)	240	
40–44	**.39 (p = .007)**	.08 (p = .588)	.04 (p = .782)	.05 (p = .738)	40	
45–49	**.33 (p = .028)**	**.30 (p = .043)**	.09 (p = .559)	.07 (p = .641)	40	
50–54	**.35 (p = .020)**	.07 (p = .672)	**.44 (p = .002)**	.13 (p = .389)	40	
55–59	.17 (p = .271)	.27 (p = .079)	.15 (p = .329)	.27 (p = .079)	40	
60–64	.12 (p = .426)	**.39 (p = .006)**	.23 (p = .132)	.09 (p = .586)	40	
65–69	.10 (p = .507)	.10 (p = .519)	.02 (p = .916)	.03 (p = .832)	40	

**C5 Pillars**						

	C4–5 IVD	C4–5 Facet	C5–6 IVD	C5–6 Facet	Sample size	

All ages	**.16 (p = .009)**	.04 (p = .499)	**.21 (p = .001)**	.03 (p = .594)	240	
40–44	.09 (p = .580)	.21 (p = .165)	**.37 (p = .012)**	.02 (p = .904)	40	
45–49	.09 (p = .559)	.10 (p = .519)	.17 (p = .273)	.01 (p = .941)	40	
50–54	**.30 (p = .047)**	.06 (p = .723)	.26 (p = .091)	.06 (p = .705)	40	
55–59	.16 (p = .292)	.17 (p = .279)	**.32 (p = .030)**	.08 (p = .604)	40	
60–64	**.36 (p = .014)**	.23 (p = .140)	.17 (p = .271)	.01 (p = .949)	40	
65–69	.10 (p = .516)	.05 (p = .726)	.02 (p = .874)	.15 (p = .324)	40	

**C6 Pillars**						

	C5–6 IVD	C5–6 Facet	C6–7 IVD	C6–7 Facet	Sample size	

All ages	**.14 (p = .024)**	.12 (p = .058)	.13 (p = .070)	.04 (p = .536)	240	
40–44	.05 (p = .751)	.07 (p = .633)	.11 (p = .490)	.16 (p = .311)	40	
45–49	.00 (p = 1.00)	.11 (p = .490)	.22 (p = .144)	.16 (p = .292)	40	
50–54	.16 (p = .311)	.06 (p = .705)	.05 (p = .751)	.12 (p = .429)	40	
55–59	**.39 (p = .008)**	.29 (p = .056)	.10 (p = .507)	.22 (p = .144)	40	
60–64	.00 (p = 1.00)	.10 (p = .525)	.10 (p = .507)	.11 (p = .490)	40	
65–69	**.35 (p = .018)**	.10 (p = .525)	.29 (p = .058)	.15 (p = .342)	40	

*IVD = Intervertebral Disc †Bold Type = statistically significant correlation coefficients

**Table 2:** Segmental association between CPH and DJD using the odds ratio (5-year age categories)

**C3 Pillars**											
	C1–2		C2–3 IVD		C2–3 F		C3–4 IVD		C3–4 F		

	OR	95% CI	OR	95% CI	OR	95% CI	OR	95% CI	OR	95% CI	Sample Size

overall	N/A	N/A	2.05	.41–10.20	1.92	.81–4.57	0.95	.31–2.92	1.29	.57–2.93	240
40–44	N/A	N/A	N/A	N/A	0.00	N/S	0.00	N/S	0.00	N/S	40
45–49	0.00	N/S	8.50	.44–163.85	7.11	.83–60.75	1.21	.11–12.81	1.00	.10–10.41	40
50–54	4.13	.30–56.38	N/A	N/A	1.94	.17–21.92	4.13	.30–56.38	0.84	.08–8.66	40
55–59	0.00	N/S	0.00	N/S	4.33	.62–30.25	0.00	N/S	1.28	.19–8.72	40
60–64	0.00	N/S	0.00	N/S	1.38	.08–23.67	0.00	N/S	2380.39	N/S	40
65–69	0.00	N/S	1.50	.14–16.32	1.05	.17–6.60	0.92	.15–5.76	3.95	.42–37.49	40

**C4 Pillars**											

	C3–4 IVD		C3–4 F		C4–5 IVD		C4–5 F				
	
	OR	95% CI	OR	95% CI	OR	95% CI	OR	95% CI	Sample Size		

overall	1.77	.66–4.76	0.64	.26–1.60	**2.81**	**1.22–6.42**	0.51	.20–1.33	240		
40–44	135482.50	N/S	1.94	.17–21.92	0.72	.07–7.34	1.50	.14–16.32	40		
45–49	7379.52	N/S	6133.63	N/S	0.00	N/S	0.00	N/S	40		
50–54	12.80	.97–168.70	1.49	.24–9.35	**18.74**	**1.95–179.83**	0.38	.04–3.61	40		
55–59	0.00	N/S	0.00	N/S	2.54	.37–17.25	0.00	N/S	40		
60–64	0.00	N/S	0.00	N/S	5.31	.50–56.39	0.57	.07–4.50	40		
65–69	2.00	.25–15.99	2.14	.20–22.65	1.12	.14–8.82	0.80	.10–6.32	40		

**C5 Pillars**											

	C4–5 IVD		C4–5 F		C5–6 IVD		C5–6 F				
	
	OR	95% CI	OR	95% CI	OR	95% CI	OR	95% CI	Sample Size		

overall	**1.90**	**1.08–3.34**	0.83	.49–1.42	**2.48**	**1.32–4.66**	0.98	.56–1.73	240		
40–44	1.50	.36–6.32	0.23	.02–2.13	**5.49**	**1.39–21.59**	0.89	.13–6.00	40		
45–49	1.56	.35–6.88	1.73	.32–9.17	2.29	.51–10.28	1.06	0.25–4.45	40		
50–54	4.00	.98–16.27	1.29	.32–5.17	3.27	.80–13.35	0.75	.17–3.33	40		
55–59	2.00	0.55–7.31	0.47	.12–1.88	8993.20	N/S	1.40	.39–5.00	40		
60–64	**5.34**	**1.35–21.14**	0.38	.10–1.40	2.33	.50–10.77	0.96	.27–3.36	40		
65–69	0.66	.19–2.31	1.25	.36–4.36	0.88	.19–4.16	1.89	.53–6.69	40		

**C6 Pillars**											

	C5–6 IVD		C5–6 F		C6–7 IVD		C6–7 F				
	
	OR	95% CI	OR	95% CI	OR	95% CI	OR	95% CI	Sample Size		

overall	**1.90**	**1.08–3.34**	1.69	.96–2.96	1.60	.96–2.67	1.21	.66–2.22	240		
40–44	1.22	.35–4.23	1.59	.24–10.70	1.62	.41–6.34	0.00	N/S	40		
45–49	1.00	.27–3.67	1.62	.41–6.34	3.05	.66–14.13	3.35	.32–35.36	40		
50–54	2.00	.52–7.72	1.33	0.30–5.92	0.82	.24–2.83	1.89	.38–9.27	40		
55–59	31431.80	N/S	3.50	.94–12.97	1.56	.42–5.76	0.33	.07–1.52	40		
60–64	1.00	.24–4.18	1.50	.43–5.25	1.56	.42–5.76	1.62	.41–6.34	40		
65–69	**10.23**	**1.12–93.34**	1.5	.43–5.25	4.83	.86–27.22	1.83	.52–6.43	40		

*IVD = Intervertebral Disc †Bold Type = statistically significant odds ratios

The strongest overall (i.e. not stratified by age categories) associations were demonstrated between C4 CPH and C4–5 DDD and between C5 CPH and C5–6 DDD: C = .16 (p = .012), OR = 2.80 (95% CI = 1.22–6.42); C = .21 (p = .001), OR = 2.48 (95% CI = 1.32–4.661), respectively. The age-stratified ORs and C-coefficients were similar to the overall levels of association, except between C4 CPH and C3–4 DDD, where the C-coefficients for the 40–54 years of age subgroups were significant (all 3 C-coefficients >.30 (p < .05)) but the overall relationship was essentially zero (C = .07; p = .252) [Table 1]. Generally speaking, however, the ORs tend to corroborate with their corresponding C-coefficients as far as association strength (effect size) and inferability (statistical significance) are concerned [Table 2].

The stratified associations of statistical significance were mostly demonstrated between C4 and C5 CPH and the adjacent level DDD, except in the oldest age subgroup (65–69 yrs) [Table 1 and Table 3]. Pillar hyperplasia at C4 and C4–5 DDD in the 50–54 years of age category demonstrated the strongest association: C = .44 (p = .002), OR = 18.74 (95% CI = 1.95–179.83) [Table 1 and Table 2].

**Table 3:** Segmental association between CPH and DJD using the odds ratio(10-year age-categories)

**C3 Pillars**											
	C1–2		C2–3 IVD		C2–3 F		C3–4 IVD		C3–4 F		
	
	OR	95% CI	OR	95% CI	OR	95% CI	OR	95% CI	OR	95% CI	Sample Size

overall	N/A	N/A	2.05	.41–10.20	1.92	.81–4.57	0.95	.31–2.92	1.29	.57–2.93	240
40–49	0.00	N/A	8.75	.50–153.81	4.79	.74–30.97	1.14	.12–10.53	0.62	.07–5.38	80
50–59	1.19	.12–11.01	0.00	N/S	2.92	.72–11.87	0.75	.08–6.67	1.07	.25–4.56	80
60–69	0.00	N/S	1.19	.19–18.78	1.00	.22–4.52	1.08	.20–5.85	4.99	.58–42.69	80

**C4 Pillars**											

	C3–4 IVD		C3–4 F		C4–5 IVD		C4–5 F				
	
	OR	95% CI	OR	95% CI	OR	95% CI	OR	95% CI	Sample Size		

overall	1.77	.66–4.76	0.64	.26–1.60	**2.81**	**1.22–6.42**	0.51	.20–1.33	240		
40–49	5.67	.85–37.56	2.86	.47–17.57	0.58	.06–5.27	1.03	.11–9.65	80		
50–59	1.50	.28–8.11	0.45	.09–2.22	**6.71**	**1.65–27.34**	0.17	.02–1.37	80		
60–69	1.08	.20–5.85	0.34	.07–1.53	2.33	.52–10.52	0.67	.16–2.91	80		

**C5 Pillars**											

	C4–5 IVD		C4–5 F		C5–6 IVD		C5–6 F				
	
	OR	95% CI	OR	95% CI	OR	95% CI	OR	95% CI	Sample Size		

overall	**1.90**	**1.08–3.34**	0.83	.49–1.42	**2.48**	**1.32–4.66**	0.98	.56–1.73	240		
40–49	1.52	.54–4.25	0.70	.20–2.51	**3.23**	**1.21–8.62**	0.87	.29–2.71	80		
50–59	**2.67**	**1.05–6.77**	0.74	.28–1.94	**4.14**	**1.23–13.90**	0.97	.38–2.45	80		
60–69	1.78	.73–4.35	0.70	.28–1.71	1.47	.50–4.30	1.35	.56–3.27	80		

**C6 Pillars**											

	C5–6 IVD		C5–6 F		C6–7 IVD		C6–7 F				
	
	OR	95% CI	OR	95% CI	OR	95% CI	OR	95% CI	Sample Size		

overall	**1.90**	**1.08–3.34**	1.69	.96–2.96	1.60	.96–2.67	1.21	.66–2.22	240		
40–49	1.11	.46–2.68	1.57	.53–4.65	2.15	.78–5.92	1.54	.24–9.75	80		
50–59	**3.77**	**1.20–11.79**	2.22	.86–5.74	1.11	.46–2.68	0.75	.26–2.15	80		
60–69	2.43	.81–7.29	1.49	.62–3.60	2.05	.84–5.04	1.70	.68–4.22	80		

*IVD = Intervertebral Disc †Bold Type = statistically significant odds ratios

Only two older subgroups demonstrated moderately-strong and statistically significant associations, and these were between C6 CPH and C5–6 DDD for the 55–59 and 65–69 yr old subgroups: C = .39 (p = .008), OR = 31431.8 (95% CI = 0.0–1.11 × 10^56^); C = .35 (p = .018), OR = 10.23 (95% CI = 1.12–93.34), respectively; but this relationship did not consistently apply to the younger subgroups. Less strong, but also statistically significant, were the relationships between C5 CPH and C4–5 DDD in the 60–64 yrs of age subgroup, and between C5 CPH and C5–6 DDD in the 55–59 yrs of age subgroup: C = .36 (p = .014), OR = 5.343 (95% CI = 1.35–21.14); and C = .32 (p = .03), OR = 8993.2 (95% CI = 0.0–8.1 × 10^38^), respectively [Table 1 and Table 2].

Several discrepancies between the two analyses (C-coefficient vs OR) occurred. Since these may have been due (in part) to underpowered age-subgroup sample sizes, the data were also analyzed using broader 10-year age categories; this helped to elevate the subgroup sample sizes towards the more desirable 'n = 80' suggested by our sample size estimate. These results are presented in Table 3. The findings here demonstrate a similar pattern to the overall OR results, with the strongest stratified association evident between C4 CPH and C4–5 DDD in the 50–59 age-category: OR = 6.71 (95% CI = 1.65–27.34). Statistically-significant associations are also evident between C5 CPH and C4–5 DDD and between C5 CPH and C5–6 DDD both overall, and in the 40–59 age-categories [Table 3]. In general, however, most associations between segmental CPH and adjacent level facet degeneration were not statistically or clinically significant.

## Discussion

The only known clinically relevant result of cervical pillar hyperplasia, as demonstrated in the literature, is its straightening effect on the cervical spine lordosis [2,3]. Therefore, with such little research on what effect cervical pillar hyperplasia may potentially have on cervical spine biomechanics, it is important to explore any possible clinical consequences of this condition. In our preliminary study [4], we assessed the possibility that altered spinal biomechanics due to CPH may lead to degenerative changes.

Our present study suggests that a generally weak-to-moderate segmental association exists between C4 and C5 CPH and adjacent level degenerative disc disease (DDD), with the strongest (overall) association demonstrated between C5 CPH and C5–6 DDD. The odds of segmental DDD occurring together with the adjacent presence of CPH for the overall age-categories are approximately two-to-one. Age-stratified results demonstrated the same pattern of association (with one exception), even reaching the initially hypothesized moderately-strong association levels of C = 0.4 and OR > 5.0, in some age categories [Table 1, 2, 3]. Pillar hyperplasia at C4 and C4–5 DDD in the 50–54 year age category had the strongest stratified association; nevertheless, generally, the segmental relationship between CPH and DJD did not reach the initially proposed association of clinical importance (C > .4 and OR > 5.0) across all age categories.

As mentioned previously, the discrepancies between the C-coefficients and ORs in the younger age subgroups (e.g. 40–54 years) for C4 CPH and C3–4 DDD may be due to sample size and/or CPH and DJD prevalence inadequacies; these, in turn, can sometimes amplify limitations of the computational formulas themselves. More specifically, naturally occurring low pillar hyperplasia prevalence at C3 and low DJD prevalence in adjacent segments, were likely the cause of the lack of statistically significant associations at those respective levels, which in turn, may be due to the likelihood that there are other known contributors to the development of DJD; these include trauma, genetic, metabolic, and inflammatory processes [18-20,23], but they were not tested in our study. Nevertheless, our results suggest that cervical pillar hyperplasia (CPH) is only weakly-to-moderately correlated with the presence of degenerative joint disease; therefore, it may contribute somewhat to the development of DJD, but the body may also compensate to some extent for the changes resulting from its slightly aberrant biomechanics. Considering that there are likely several clinically important contributing factors leading to the development of degenerative joint disease, hyperplasia may be but one of several of these. Our findings, when subjected to *Coefficient of Determination *analysis, suggest that at least in some cervical-spine levels, and in some age categories, CPH may contribute to approximately 9–18% (p ≤ .05) of the development of DJD [35].

Some limitations encountered in this study were poor visualization of C2–3 when assessing films for CPH and DJD, poor visualization of C1–2 while assessing for DJD, and the sometimes inconsistent presence of hyperplasia in the right and left pillar at a single cervical level. Due to diverging rays of the x-ray beam and small variances in patient positioning, the two articular pillars at any particular level may not be perfectly superimposed on the lateral cervical radiograph, thus allowing the evaluation of each pillar separately for CPH. We were also limited by the lack of any peer-reviewed literature confirming the reliability of evaluating DJD severity. Another potential assessment bias in the present study was the fact that one of the assessors, an experienced radiologist, could not be blinded to the presence/absence of cervical pillar hyperplasia while assessing for DJD. A future study could eliminate this bias by blinding the assessing radiologist to the purpose of the study.

Another limitation is that the OR sample size estimate was performed *ex post facto *(after the data were collected), and this, as well as the data analysis (the hypothesis testing part) itself, revealed that the sample sizes in most of the age-specific categories were too small to yield adequate (≥80%) power i.e. statistical significance. Age categories were combined in an attempt to compensate for this, but future studies should endeavour to collect larger samples.

## Conclusion

Our study suggests that cervical pillar hyperplasia (CPH) is weakly-to-moderately associated with the presence of degenerative joint disease (the DDD component of DJD); more specifically, an approximate two-to-one odds, or a weak-to-moderate association exists between C4 and C5 CPH and adjacent level degenerative disc disease (DDD), with the strongest (overall) associations occurring between C4 CPH and C4–5 DDD and between C5 CPH and C5–6 DDD. *Coefficients of Determination *of 0.09–0.18 suggest that at some cervical-spine levels, and in some age categories, CPH may contribute to approximately 9–18% (p ≤ .05) of the development/etiology of DJD. Therefore, while CPH may be but one of many contributing factors to the development of cervical-spine degeneration, chiropractic clinicians, who are actively treating patients, need to be aware of all conditions, including CPH, that may influence their patients' clinical presentations, susceptibility/response to available treatments, and their prognostic factors. Finally, we also hypothesize that the correlations between level-specific CPH and DJD may be more meaningful when the *severity *of DJD is also factored into the analysis, and we therefore recommend that future research consider this.

## Competing interests

The author(s) declare that they have no competing interests.

## Authors' contributions

MS participated in the design of the study, the acquisition, analysis and interpretation of data and she drafted the manuscript.

CP conceived of the study concept, participated in the acquisition of the data and helped to draft the manuscript.
